# Incidence and predictors of tuberculosis among HIV-infected adults after initiation of antiretroviral therapy in Nigeria, 2004-2012

**DOI:** 10.1371/journal.pone.0173309

**Published:** 2017-03-10

**Authors:** Ishani Pathmanathan, E. Kainne Dokubo, Ray W. Shiraishi, Simon G. Agolory, Andrew F. Auld, Dennis Onotu, Solomon Odafe, Ibrahim Dalhatu, Oseni Abiri, Henry C. Debem, Adebobola Bashorun, Tedd Ellerbrock

**Affiliations:** 1 Division of Global HIV and TB, U.S. Centers for Disease Control & Prevention, Atlanta, GA, United States of America; 2 Epidemic Intelligence Service, U.S. Centers for Disease Control & Prevention, Atlanta, GA, United States of America; 3 Division of Global HIV and TB, U.S. Centers for Disease Control & Prevention, Abuja, Nigeria; 4 Department of Nursing, University of South Alabama, Mobile, AL, United States of America; 5 HIV/AIDS Division, Federal Ministry of Health, Abuja, Nigeria; National and Kapodistrian University of Athens, GREECE

## Abstract

**Background:**

Nigeria had the most AIDS-related deaths worldwide in 2014 (170,000), and 46% were associated with tuberculosis (TB). Although treatment of people living with HIV (PLHIV) with antiretroviral therapy (ART) reduces TB-associated morbidity and mortality, incident TB can occur while on ART. We estimated incidence and characterized factors associated with TB after ART initiation in Nigeria.

**Methods:**

We analyzed retrospective cohort data from a nationally representative sample of adult patients on ART. Data were abstracted from 3,496 patient records, and analyses were weighted and controlled for a complex survey design. We performed domain analyses on patients without documented TB disease and used a Cox proportional hazard model to assess factors associated with TB incidence after ART.

**Results:**

At ART initiation, 3,350 patients (95.8%) were not receiving TB treatment. TB incidence after ART initiation was 0.57 per 100 person-years, and significantly higher for patients with CD4<50/μL (adjusted hazard ratio [AHR]: 4.2, 95% confidence interval [CI]: 1.4–12.7) compared with CD4≥200/μL. Patients with suspected but untreated TB at ART initiation and those with a history of prior TB were more likely to develop incident TB (AHR: 12.2, 95% CI: 4.5–33.5 and AHR: 17.6, 95% CI: 3.5–87.9, respectively).

**Conclusion:**

Incidence of TB among PLHIV after ART initiation was low, and predicted by advanced HIV, prior TB, and suspected but untreated TB. Study results suggest a need for improved TB screening and diagnosis, particularly among high-risk PLHIV initiating ART, and reinforce the benefit of early ART and other TB prevention efforts.

## Introduction

Tuberculosis (TB) and HIV are the two leading causes of death from infectious diseases worldwide, and are often co-existing. Despite improvements in preventive, diagnostic and treatment capabilities, TB remains the leading cause of morbidity and mortality among persons living with HIV (PLHIV), who represented a quarter of the 1.5 million global TB deaths estimated to have occurred globally in 2014 [1–2]. Efforts have therefore been increasingly focused on addressing this dual burden of disease through collaborative TB/HIV activities, as outlined in World Health Organization (WHO) policy and guidelines [3–4]. Key interventions recommended to reduce the TB burden among PLHIV include intensified TB case-finding and treatment, isoniazid preventative therapy (IPT), TB infection control, and early antiretroviral therapy (ART) initiation [4–6].

Nigeria is the most populous country in sub-Saharan Africa, with a high burden of both TB and HIV [2]. In 2015, it had the second highest number of HIV cases and the highest number of AIDS-related deaths in the world, with 3.5 million PLHIV (representing a national prevalence of 3.1% among adults aged 15–49) and 180,000 AIDS-related deaths [7–10]. In addition, it ranked sixth in terms of the absolute number of patients estimated to have TB and fourth in terms of TB incidence among the 22 high burden countries accounting for 80% of the total global burden of TB, with an estimated 590,000 prevalent cases (0.3%) and a rate of 322 incident cases per 100,000 people per year. TB and HIV co-infection is common, with 100,000 incident TB cases and 78,000 TB deaths reported among PLHIV in 2014 [2]. To address this dual disease burden the Nigerian Ministry of Health has scaled up HIV care and treatment – including specific TB reduction strategies – with support from the United States President’s Emergency Plan for AIDS Relief (PEPFAR).

According to Nigerian and WHO clinical guidelines, adults and adolescents presenting for HIV care should be screened for TB symptoms initially and at each subsequent encounter using at least a four-symptom clinical algorithm [4–5]. Patients without active TB should be provided IPT to prevent later activation of presumed latent infection, while a positive TB screen should prompt further diagnostic workup. TB diagnosis is often complicated by immunocompromise in many PLHIV, who are more likely to have smear-negative or extra-pulmonary TB [4]. While treatment of PLHIV with ART has been shown to reduce future incidence of TB, risk remains especially high in the immediate period following ART initiation due to unmasking of previously undiagnosed disease during immune reconstitution as well as new, potentially nosocomial infection. Unmasked symptoms during this early time period often occur more rapidly and with greater severity, complicating early treatment and resulting in increased morbidity and mortality as well as potential threats to infection control [11–13]. Given this concern, WHO has prioritized research efforts that address improving screening for subclinical and extra-pulmonary TB among PLHIV, identifying the true prevalence, natural history, and importance of subclinical TB in this population, and pinpointing potential risk factors and predictors of TB and TB Immune Reconstitution Inflammatory Syndrome (IRIS) that can be targeted through programmatic change [14]. In Nigeria, although almost 750,000 adults and children were reported to be receiving ART by 2014, the incidence of TB in these patients after ART initiation has not been estimated [7].

The main objective of this study was to examine the incidence and determinants of TB after ART initiation in Nigerian adults not on TB treatment at baseline. Multiple prior studies have looked at this relationship in high-income countries, with a large study in Europe and North America noting a TB incidence of 1.3 cases per 100 person-years (PY) within 90 days of ART initiation that was associated with higher baseline prevalence of TB and degree of initial immunosuppression [15]. Subsequent studies in sub-Saharan Africa have demonstrated incidence rates ranging from 0.9 to 7.8 per 100 PYs, as described in a recent meta-analysis [16], although a study conducted in one teaching hospital in Nigeria showed a lower rate of 0.8 per 100 PYs [17]. TB incidence after ART initiation in all these settings was frequently cited as highest within the first few months of treatment and associated with suboptimal ART adherence, age, male gender, low weight (less than 60 kilograms), anemia, degree of baseline immunodeficiency (CD4<50/μL), and/or sub-optimal immune reconstitution while on ART [18–22].

We conducted a retrospective cohort study among a nationally representative sample of adults in HIV care and treatment programs in Nigeria between 2004 and 2012, to better characterize the patient population and determine factors associated with incident TB after ART initiation.

## Methods

### Study design

This is a secondary analysis of retrospective cohort data from the Nigeria ART Outcomes Study, which was designed to assess ART outcomes and retention in care during a period of rapid scale-up of Nigeria’s HIV care and treatment program. Detailed study methods have been previously described [23].

### Study population and sampling

The study population was drawn from a nationally representative sample of adults 15 years or older who were enrolled in PEPFAR-supported care and treatment programs in Nigeria between January 2004 and December 2012. All 139 supported healthcare facilities enrolling 50 or more patients on ART by August 31, 2009 were eligible, representing 78% of the total number of sites supported by PEPFAR to provide ART services in the country (which themselves represented over 85% of Nigerian government facilities). Of these, 35 sites were selected for review using probability-proportional-to-size sampling. Size was based on the number of patients ever enrolled on ART by December 31, 2012. Participant inclusion criteria were age greater or equal to 15 years at the time of ART initiation at a selected site, and ART initiated on or after January 1, 2004 and at least 12 months prior to the time of data abstraction. Medical records of eligible ART patient registers at selected care and treatment facilities were randomly selected, for a total of 3,496 participants.

### Exposure and outcome variables

Incident TB after ART initiation was defined as a new clinically diagnosed or bacteriologically-confirmed (with a biologic specimen positive for *Mycobacterium tuberculosis*) case of TB, documented for PLHIV not taking TB treatment when they began ART. Patients were considered to have prevalent TB only if they were documented to be on TB treatment at baseline (as is the convention for TB diagnosed before ART initiation), and were excluded from subsequent incidence analysis. Patients were classified as having suspected TB (now more appropriately termed “presumptive TB”) if they had at least one TB symptom but initiated ART before or in the absence of a TB diagnostic workup. Since our objective was to assess TB incidence in patients not on TB treatment when they began ART, these patients were included in our sub-population analysis. Based on WHO and Nigerian guidelines at the time, ART should not have been started in patients with presumed active TB until eight weeks after TB treatment initiation, or two weeks after TB treatment initiation for patients with profound immunosuppression (CD4<50/μL).

Based on a literature review, variables assessed as potential risk-factors for incident TB included year of ART initiation, age, gender, weight, employment and marital status, education level, baseline CD4 count, WHO stage of HIV disease, and functional status [16–22].

### Data analysis

Abstracted data were analyzed using Stata 13.1^**®**^ (StataCorp. 2013. *Stata Statistical Software*: *Release 13*. College Station, TX: StataCorp LP), weighted to the national ART patient population and controlled for a complex survey design. A domain analysis was performed on a subgroup of patients not on TB treatment at baseline, as described above. Multiple imputation with chained equations was used to impute missing data for variables of interest, assuming that they were missing at random (MAR) and that all patients had complete time-to-event data including an ART initiation date and, if they developed TB, a TB incidence date. Patients not documented as being newly diagnosed with TB during follow-up were censored at their last visit before December 2012 or at their date of death or transfer out if these events occurred. A multivariable Cox proportional hazards regression model was used to estimate hazard ratios adjusted for all covariates and 95% confidence intervals (CI) for factors associated with TB incidence. Some non-proportionality was identified with gender, and so subsequent models were stratified by this variable. Kaplan-Meier survival curves were used to assess cumulative probability of remaining TB-free over time, stratified by baseline variables.

### Ethics statement

Study methods were approved by the Institutional Review Board at the United States Centers for Disease Control and Prevention and the Nigerian Health Research Ethics Committee. Informed consent was waived because patient record review and data abstraction were considered to be routine, anonymous, and of minimal risk to subjects.

## Results

### Patient characteristics

**Fig 1:**
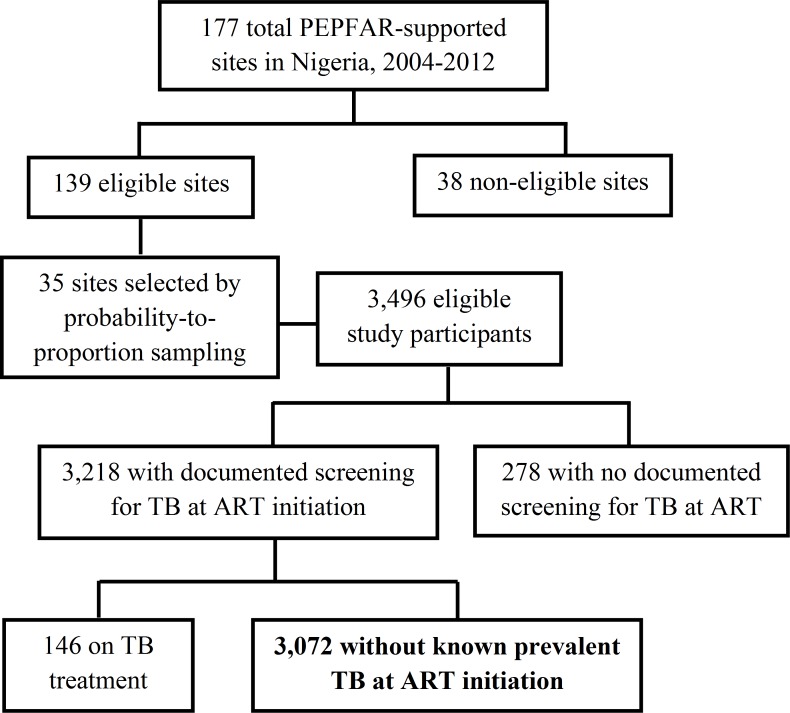
Participant Flow Diagram (Unweighted Study Sample Population).

Data were abstracted and analyzed from medical records of 3,496 eligible adult ART patients enrolled on ART between 2004 and 2012. Fig 1 describes the unweighted sample population.

Results weighted to the national ART patient population indicate that 92% (95% CI 91.1–92.9%) of patients initiating ART were screened for TB, of which only 4.2% (95% CI 3.1–5.4%) were already on TB treatment. Of those without known prevalent TB at ART initiation, 1.2% (95% CI 0.4–2.0%) reported prior TB, and 7.0% (95% CI 4.6–9.5%) were documented as having suspected TB, indicating a positive TB symptom screen. Only 0.5% (95% CI 0.1–0.9%) of patients eligible for IPT (without suspected or prior TB and not on TB treatment) were on IPT at ART initiation.

Demographic and clinical characteristics of patients without known prevalent TB at ART initiation are shown in Table 1. Population estimates between original and imputed data were generally similar across all variables. Patients on TB treatment at baseline were excluded from the sub-population analysis, however population estimates between the excluded group and the sample sub-population were also largely similar, with the exception that a greater proportion of the excluded group had a low CD4 count, an advanced stage of HIV disease, and poor functional status.

**Table 1:** Patient Characteristics, by Known Prevalence of Active TB at ART Initiation.

All Patients at Enrollment N = 3,496)				TB Treatment at Enrollment (n = 158)	No Treatment at Enrollment (n = 3,338)	
	Original		Imputed			
	N*	% [CI]	% [CI]	% [CI]	% [CI]	p
**ART Start Year** (n = 3,481)						
2004–2006	402	15.1 [9.7–22.6]	15.1 [8.7–21.4]	18.5 [4.7–32.4]	14.9 [8.6–21.2]	0.907
2007–2009	1,953	55.4 [51.0–59.7]	55.4 [51.0–59.8]	49.6 [39.6–59.6]	55.7 [51.3–60.0]	
2010–2012	1,126	29.5 [24.5–35.1]	29.5 [24.2–34.9]	31.9 [20.7–43.1]	29.4 [24.0–34.9]	
**Enrollment Age** (n = 3,479)						
15–24	390	10.6 [9.1–12.3]	10.6 [9.0–12.2]	8.9 [4.1–13.7]	10.7 [9.1–12.3]	0.350
25–34	1,488	42.4 [40.2–44.7]	42.4 [40.2–44.7]	41.1 [32.6–49.6]	42.5 [40.2–44.8]	
35–44	1,045	30.6 [28.7–32.6]	30.6 [28.7–32.5]	31.7 [24.5–39.0]	30.6 [28.6–32.5]	
45+	556	16.3 [14.9–17.8]	16.3 [14.8–17.8]	18.2 [11.1–25.2]	16.2 [14.7–17.7]	
**Gender** (n = 3,496)						
Female	2,320	65.9 [63.1–68.6]	65.9 [63.2–68.7]	60.6 [52.7–68.4]	66.2 [63.3–69.1]	0.183
Male	1,176	34.1 [31.3–36.9]	34.1 [31.3–36.8]	39.4 [31.6–47.5]	33.8 [30.9–36.7]	
**Marital Status** (n = 3,336)						
Single/Divorced/Widowed	1,164	36.4 [33.3–39.5]	36.3 [33.3–39.5]	42.7 [32.1–53.4]	36.1 [32.9–39.2]	0.193
Married	2,172	63.6 [60.5–66.7]	63.6 [60.5–66.7]	57.3 [46.6–67.9]	63.9 [60.8–67.1]	
**Education Level** (n = 2,836)						
None	355	12.3 [7.3–20.1]	12.9 [6.8–19.0]	12.3 [5.3–19.3]	12.9 [6.7–19.1]	0.419
Primary	685	23.7 [20.7–26.8]	23.5 [20.3–26.7]	29.0 [20.5–37.6]	23.2 [20.1–26.4]	
Secondary	1,173	42.1 [38.3–46.1]	41.4 [37.6–45.3]	39.3 [29.4–49.1]	41.5 [37.5–45.6]	
Tertiary	623	21.8 [18.4–25.7]	22.2 [18.3–26.1]	19.4 [9.5–29.4]	22.3 [18.4–26.2]	
**Employment** (n = 3,076)						
Employed	1,797	62.6 [55.0–69.6]	61.1 [53.3–68.8]	61.8 [51.2–72.4]	61.0 [53.2–68.8]	0.864
Unemployed	1,279	37.4 [30.3–45.0]	38.9 [31.2–46.7]	38.2 [27.6–48.8]	39.0 [31.2–46.8]	
**Baseline CD4** (n = 2,848)						
≥ 200/μL	1,054	36.4 [32.8–40.1]	37.1 [33.3–40.9]	28.9 [17.8–40.1]	37.5 [33.7–41.2]	**0.035**
50- < 200/μL	1,365	48.7 [45.4–51.9]	48.2 [45.1–51.3]	46.7 [36.9–56.4]	48.3 [45.2–51.4]	
< 50/μL	429	15.0 [12.6–17.6]	14.6 [12.2–17.1]	24.4 [14.8–34.0]	14.2 [11.9–16.6]	
**Baseline Weight** (n = 3,272)						
<45kg	404	12.1 [10.0–14.7]	12.7 [10.0–15.3]	16.4 [8.3–24.5]	12.5 [9.8–15.2]	0.173
45-60kg	1,716	51.2 [47.8–54.6]	50.7 [47.5–54.0]	53.6 [46.2–61.0]	50.6 [47.2–54.0]	
>60kg	1,152	36.6 [32.1–41.4]	36.6 [32.0–41.3]	30.0 [20.8–39.2]	36.9 [32.2–41.7]	
**WHO Stage** (n = 3,264)						
Stage I	845	25.8 [20.8–31.5]	26.1 [20.6–31.6]	9.6 [4.7–14.6]	26.8 [21.2–32.5]	**0.000**
Stage II	850	26.8 [23.1–30.9]	26.9 [23.0–30.8]	13.2 [6.1–20.4]	27.5 [23.6–31.5]	
Stage III/IV	1,569	47.4 [40.7–54.1]	47.0 [40.4–53.6]	77.1 [69.0–85.3]	45.6 [38.9–52.4]	
**Functional Status** (n = 3,160)						
Asymptomatic	2,151	64.4 [54.5–73.2]	62.7 [53.1–72.2]	46.6 [33.7–59.4]	63.4 [53.7–73.1]	**0.001**
Symptoms, normal activity	909	31.8 [24.0–40.8]	32.9 [24.5–41.3]	40.2 [28.3–52.1]	32.6 [24.0–41.2]	
Bedridden at all	100	3.8 [2.4–5.9]	4.4 [2.3–6.5]	13.3 [2.6–24.0]	4.0 [2.2–5.9]	
**Initial TB Status** (n = 3,218)						
No TB	2,789	87.5 [84.7–89.8]	87.4 [84.8–89.9]	0	91.2 [88.7–93.8]	-
On INH	16	0.4 [0.2–1.0]	0.5 [0.1–0.9]	0	0.5 [0.1–0.9]	
Suspected TB	226	6.7 [4.7–9.6]	6.7 [4.4–9.1]	0	7.0 [4.6–9.5]	
Prior TB	41	1.2 [0.6–2.3]	1.2 [0.4–1.9]	0	1.2 [0.4–2.0]	
On TB Treatment	146	4.2 [3.2–5.4]	4.2 [3.1–5.4]	100	0	

*all data weighted to the national population except ‘N’ values, which represent the original sample population

Prevalence of treated TB at baseline was 4.2% (95% CI: 3.1–5.4). Of the patient sub-population not receiving TB treatment at baseline, median age was 34 years (interquartile range [IQR]) 28–40), 66% were female (95% CI: 63–69), 64% were married (95% CI: 61–67), 87% had at least a primary school education (95% CI: 81–93), 61% were employed (95% CI 53–69), 46% had WHO stage three or four HIV disease (95% CI: 39–52), and median CD4 cell count was 161/μL (IQR 82–251) (Table 1).

### TB incidence

TB incidence weighted to the general population was 0.57 per 100 PY. Ninety-three percent (95% CI: 78–98%) of those with incident TB were estimated to still be on ART by the end of their follow-up period.

**Fig 2:**
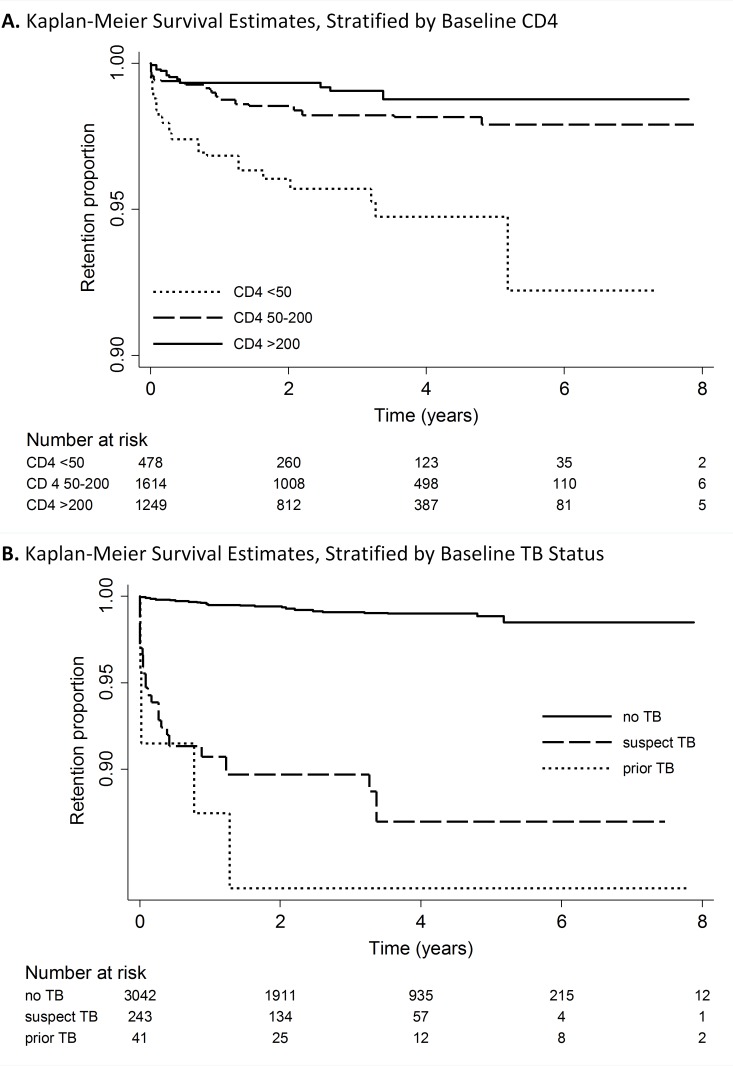
**Kaplan Meier Survival Analysis of TB-Free Survival, Stratified by Baseline: A. CD4 count; B. TB status.** (Survival analysis run on imputation #1 of 20).

Cox proportional hazard ratios of patient characteristics associated with TB incidence from the unadjusted and adjusted models are presented in Table 2. TB incidence was statistically significantly higher for patients with baseline CD4 cell count less than 50/μL (adjusted hazard ratio [AHR]: 4.2, 95% CI: 1.4–12.7) compared with patients with a baseline CD4 greater or equal to 200/μL. A Kaplan-Meier curve showing TB-free survival stratified by baseline CD4 cell count is shown in Fig 2A. A protective effect was also found for patients aged 35–44 years (AHR: 0.3, 95% CI: 0.1–0.7) compared with those aged 15–24 years. No statistically significant associations were observed for employment or marital status, education level, WHO stage of HIV disease, or baseline weight.

**Table 2:** Patient Characteristics Associated with TB Incidence after ART Initiation.

	Original	Crude				Adjusted		
	N	Rate/100	HR	95%CI	p	AHR	95% CI	p
**Year of ART Initiation**								
2004–2006	402	0.60	1.00			1.00		
2007–2009	1,953	0.50	0.48	0.29–0.80	**0.007**	0.45	0.20–1.01	0.052
2010–2012	1,126	0.83	0.55	0.25–1.23	0.143	0.62	0.29–1.35	0.218
**Age at Enrollment**								
15–24	390	0.60	1.00			1.00		
25–34	1,488	0.67	1.30	0.42–4.01	0.637	0.90	0.37–2.23	0.822
35–44	1,045	0.49	0.67	0.18–2.55	0.550	0.27	0.10–0.74	**0.013**
45+	556	0.49	0.65	0.14–2.96	0.565	0.26	0.06–1.18	0.078
Gender*								
Female	2,320	0.47	1.00					
Male	1,176	0.78	1.76	0.83–3.73	0.136	-	-	-
**Marital Status**								
Single/Divorced/Widowed	1,164	0.54	1.00			1.00		
Married	2,172	0.59	1.11	0.64–1.92	0.695	1.58	0.68–3.63	0.274
**Education Level**								
None	355	0.62	1.00			1.00		
Primary	685	0.76	2.31	0.52–10.31	0.264	1.41	0.27–7.34	0.674
Secondary	1,173	0.63	1.60	0.37–6.92	0.517	0.92	0.20–4.21	0.909
Tertiary	623	0.29	0.92	0.17–4.96	0.917	0.62	0.11–3.42	0.575
**Employment Status**								
Employed	1,797	0.59	1.00			1.00		
Unemployed	1,279	0.55	0.88	0.33–2.34	0.797	0.93	0.29–2.96	0.895
**Baseline CD4 count**								
≥ 200/μL	1,054	0.29	1.00			1.00		
50- < 200/μL	1,365	0.53	1.58	0.68–3.66	0.276	1.34	0.55–3.25	0.499
< 50/μL	429	1.62	4.89	1.51–15.84	**0.010**	4.17	1.36–12.72	**0.014**
**Baseline Weight**								
<45kg	404	0.69	1.00			1.00		
45-60kg	1,716	0.66	0.83	0.26–2.64	0.741	0.96	0.26–3.60	0.952
>60kg	1,152	0.44	0.48	0.14–1.72	0.249	0.80	0.21–3.01	0.727
**WHO Stage**								
Stage I	845	0.30	1.00			1.00		
Stage II	850	0.64	2.58	0.86–7.70	0.087	1.88	0.64–5.48	0.238
Stage III/IV	1,569	0.72	2.17	0.77–6.12	0.137	0.62	0.28–1.68	0.337
**Functional Status**								
Asymptomatic	2,151	0.36	1.00			1.00		
Symptoms but normal activity	909	0.92	2.24	0.90–5.59	0.082	1.76	0.76–4.08	0.174
Bedridden at all	100	1.49	4.79	0.93–24.68	0.060	2.75	0.52–14.55	0.215
**TB Status at ART Initiation**								
No TB	2,789	0.27	1.00			1.00		
Suspected TB	226	4.52	11.2	5.47–22.75	**0.000**	12.23	4.46–33.50	**0.000**
Prior TB	41	3.94	16.3	3.23–82.16	**0.001**	17.64	3.54–87.88	**0.001**

* Some non-proportionality was identified with gender and so subsequent models were stratified by this variable

Patients classified as having suspected TB at baseline but who were not diagnosed with or treated for TB were more likely to develop incident TB during follow up than those classified as having no TB (AHR: 12.2, 95% CI: 4.5–33.5), as were patients with a prior history of TB disease (AHR: 17.6, 95% CI: 3.5–87.9) (Table 2). For patients with a prior history of TB, a new diagnosis of TB frequently occurred soon after ART initiation, with 9.4% of this group recorded as developing TB at a discrete follow-up point within six months, and a total of 14.2% were reported to have incident TB a little over one year from ART initiation. In the suspected TB group, 4.9% were recorded as having incident TB by six months, 6.5% by one year, and 8.5% by three years after ART initiation (Fig 2B).

None of the patients who were receiving IPT at baseline developed incident TB during follow-up.

## Discussion

This study provides insight into TB diagnosis and care for HIV-infected patients along a continuum of care in Nigeria. Ninety two percent of patients beginning ART had documented screening for TB which is encouraging. Current WHO recommendations call for all adult PLHIV to be routinely screened for TB at every clinical encounter using, at a minimum, a standard four-symptom screen of cough, fever, night sweats, and weight loss [3]. However, prevalent TB at the time of ART initiation (defined as being on TB treatment) was observed to be much lower in this cohort (4.2%) than in prior studies in countries with a similar TB prevalence in the general population, which have documented TB prevalence rates at ART initiation ranging from 11–31.5% [11,15,18,24–27]. This may indicate that, although screening occurs routinely in Nigerian health facilities, it may not detect all patients with TB. Some of the cited studies from South Africa used culture or other diagnostic methods for TB screening, whereas the patients in this study were typically screened using only a four-symptom screen. In addition, the observed incidence of TB in Nigerian adults after starting ART was found to be lower in this study (0.57 cases per 100 PY) than in prior studies conducted among adults in sub-Saharan Africa, which have described a range of 0.9–7.9 cases per 100 PY [15–16,18–22]. This low observed rate may be due to exclusion of patients with TB at baseline in the sub-population analysis which, while necessary for measuring time-to-event data, excluded a population known to contribute to TB incidence after ART initiation. However, the number of people considered to have prevalent TB and thus excluded from analysis was low. In addition, our findings are corroborated by a single-site analysis from Nigeria which found a similarly low incidence rate in their cohort, as well as a national cross-sectional population-based survey in 2012 that found a prevalence of bacteriologically-confirmed TB much higher than previous estimates based on case notification data, indicating widespread suboptimal prior TB case detection in the country [2,17,28]. Per this survey, the estimated TB case detection rate in Nigeria was only 16% [2,28]. The low observed prevalence and incidence rates of TB in this study cohort suggests systematic failure to diagnose TB in this patient population at baseline and/or to diagnose or document TB in HIV clinic registers at subsequent clinical encounters. These observations warrant further evaluation of nation-wide compliance with TB symptom screening guidelines, referral, and documentation practices within ART clinic settings and suggest a role for diagnostic tools to be incorporated into the Nigerian TB screening algorithm for HIV-infected patients.

Development of incident TB after starting ART was significantly associated with degree of immunodeficiency, which is consistent with previous findings in the literature, and reinforces the known benefit in terms of TB morbidity of early HIV diagnosis and treatment. In addition, older patient age (35–44 vs. 15–24) was protective for TB incidence. Poorer HIV treatment outcomes have been found previously among adolescents in resource-limited settings − often in conjunction with decreased adherence to ART and higher rates of loss to follow-up − which underscores the importance of specifically-designed adolescent-friendly services and interventions to improve outcomes in this traditionally high-risk population [29–31].

The association of incident TB with a prior history of TB has been shown previously, and underscores the need for increased vigilance in this particular patient population. As shown by the steep drop off in TB-free survival noted for this group immediately after ART initiation (Fig 2B), for at least 9.4% of these patients their incident TB likely either represented a delay in prevalent TB diagnosis until shortly after ART initiation, or TB IRIS. A similar though less dramatic pattern is seen for those classified as having suspected TB at baseline. One potential reason for TB diagnostic delay for PLHIV in Nigeria is limited integration of services and referral between HIV and TB clinics. However if ART was started before TB diagnosis and treatment in patients with suspected TB disease, these patients were mismanaged, as WHO and Nigerian guidelines at that time recommended initiating treatment for active TB two to eight weeks before initiating ART to reduce the risk of TB-associated IRIS [3–4,32–33]. Therefore, although some of these patients classified as developing incident TB in our model may have actually had prevalent TB at ART initiation, we intentionally included them in our analysis to highlight that these were people with a higher risk of developing TB after being started on ART, and an opportunity was missed to initiate them on TB treatment first.

The 2008 Nigerian and most recent WHO guidelines also recommend offering IPT to eligible HIV-positive patients who are not thought to have active TB disease upon routine screening [3–5]. Despite this recommendation, only 16 patients in our un-weighted study population (0.5% of those screened for TB) were documented to be on IPT at baseline. While the fact that this study began in 2004 could partially explain the poor IPT uptake observed, recent data from WHO unfortunately indicates that Nigeria still lags in this regard, with only 3.5% of PLHIV newly enrolled in HIV care estimated to be started on IPT in 2013 [34]. Interestingly, none of the study patients on IPT developed incident TB during follow-up, although our study was not adequately powered to extrapolate this finding to the national population. However, this observation reinforces the well-established importance of offering IPT to eligible HIV-positive patients without active TB, and underscores the need for significant scale-up of this practice in Nigeria.

The major strength of this study is that it drew on a large and nationally representative sample from a country with a substantial burden of TB and HIV. Nevertheless, it does have several limitations. Statistical power to detect some risk factors for TB incidence was limited by the surprisingly low overall incidence rate of TB in our ART patient cohort. Data were limited to what was available in paper ART registers and what was collected to meet the objectives of the original study; for this secondary analysis, this means that TB outcome was recorded as ‘TB” or “no TB” with a date of diagnosis, but information on specifics, such as laboratory confirmation results were not collected. Baseline TB status was recorded similarly. Missing data on baseline covariates were imputed based on the assumption that they were MAR, which may have introduced non-differential measurement error. Finally, findings regarding TB incidence are not representative of those with active TB at baseline because these patients were excluded from our sub-population analysis.

Overall, results from this study in Nigeria demonstrate an unexpectedly low reported TB prevalence at and incidence after ART initiation, as well as incident TB associated with increasing immunocompromise, prior TB, and suspected but unconfirmed and untreated TB. These findings warrant further investigation into the degree and timing of TB symptom screening, diagnosis, treatment, and documentation that is occurring at ART clinics nationwide in Nigeria. They suggest a need for improved screening and diagnosis among particularly high-risk PLHIV initiating ART, such as those with low CD4 counts. Finally, our results reinforce known recommendations to diagnose and treat HIV early, to provide IPT for those PLHIV without active TB at baseline, and to scale-up intensified TB case-finding efforts in all patients initiating ART, especially in those at advanced stages of HIV disease or for whom there is a high-suspicion of TB, so that they can be treated for TB before they undergo immune reconstitution on ART.

## Supporting information

S1 File Nigeria ART Evaluation Data Abstraction Tool Final 19 Nov 2012. (PDF)

S2 File Minimal Dataset. (DTA)
