# Supplementary material for: Incidence and predictors of tuberculosis among HIV-infected adults after initiation of antiretroviral therapy in Nigeria, 2004-2012
Source: PLoS One. 2017 Mar 10;12(3):e0173309. doi: 10.1371/journal.pone.0173309 (PMC5345814; doi:10.1371/journal.pone.0173309)
Supplement: S1 File — (PDF) [file pone.0173309.s001.pdf]

Nigeria ART Program Evaluation Data Extraction Tool

Adult Study ID:A __ __ __ **Name of Hospital/Clinic: ____________________**

Abstractor's Name: ______________________ **Date of Abstraction: D D / M M / Y Y**

A. PERSONAL INFORMATION	
1. Sex:	☐ **Male** ☐ **Female**
2. Date of Birth:	D D / M M / Y Y \
3. Age at and date of enrollment in ART program:	D D / M M / Y Y ____**Years** (cannot be missing)
4. Marital status of patient at the time of enrollment in ART program:	☐ **Single** ☐ **Married** ☐ **Divorced** ☐ **Widowed**<br>☐ **Other, specify: _______** ☐ **Missing**
5. Partner/spouse HIV status (through the most recent visit):	☐ **HIV positive** ☐ **HIV negative** ☐ **Missing/Unknown**
6. Patient education level at time of enrollment in ART program:	☐ **None** ☐ **Primary school** ☐ **Secondary school**<br>☐ **Post Secondary** ☐ **University**<br>☐ **Other, specify: ___________** ☐ **Missing**
7. Patient employment status at the time of enrollment in ART program:	☐ **Employed**<br>☐ **No, not currently employed** ☐ **Missing**
8. Was patient pregnant at time of enrollment in ART program?	☐ **Yes** ☐ **No** ☐ **N/A (male)** ☐ **Missing**
9. Did patient start ART at a different clinic BEFORE transferring into this clinic?	☐ **No** *(skip to question 12)* ☐**Yes,**<br>**if yes:** ***date of transfer in:*____/_____/_______** ☐**Missing**
10. If patient is a "**transfer in**", enter the dates of HIV ART initiation at previous facility *(if available).*	**ART Initiation:** D D / M M / Y Y ☐ **Missing**
11. Name of the ART regimen initiated at previous facility?	________________/___________________/_________________ ☐ **Missing**
**B. CLINICAL INFORMATION**	
12. Date of first confirmed HIV Positive test:	D D / M M / Y Y ☐ **Missing**
13. HIV type:	☐**HIV1** ☐**HIV2** ☐**HIV1 and 2** ☐ **Type unknown** ☐**Missing**
14. Patient height:	**_____ Meters** ☐ **Missing**
15. Patient weight at start of ART:	**__________ kg** ☐ **Missing**
16. Date medically eligible for ART:	D D / M M / Y Y ☐ **Missing**
17. Date ART started:	D D / M M / Y Y (cannot be missing)
18. Eligibility Criteria for ART:	☐ **Clinically only** ☐ **CD4** ☐ **Total Lymphocytes Count**

(check all that apply)	☐ **Missing** ☐ **Other, specify______________**
19. CD4 at start of ART:	_____ **cells/mm**^{3} ***date of test,*** D D / M M / Y Y ☐ **Missing**
20. Clinical stage at start of ART:	☐ **Stage I** ☐ **Stage II** ☐ **Stage III** ☐ **Stage VI** ☐ **Missing**
21. Functional status at start of ART:	☐ **Asymptomatic (working)** ☐ **Symptomatic normal activity (ambulatory)**<br>☐ **Bed ridden <50% of day in last month**<br>☐ **Bed ridden >50% of day in last month**<br>☐ **Missing**
22. TB status at start of ART:	☐ **No TB** ☐ **On INH prophylaxis**<br>☐ **Suspected TB**<br>☐ **Prior history of TB treatment** ☐ **On TB treatment** ☐ **Missing**
23. Opportunistic Infections (OIs) **during** ART treatment and dates of diagnosis (dx): *(check all that apply)*	☐ **Chronic diarrhea** — ***date of dx,*** D D / M M / Y Y<br>☐ **Tuberculosis (pulm/extrapulm)** — ***date of dx,*** D D / M M / Y Y<br>☐ **PCP** — ***date of dx,*** D D / M M / Y Y<br>☐ **Cryptococcosis** — ***date of dx,*** D D / M M / Y Y<br>☐ **Kaposi Sarcoma** — ***date of dx,*** D D / M M / Y Y<br>☐ **Herpes Zoster** — ***date of dx,*** D D / M M / Y Y<br>☐ **Vaginal thrush** — ***date of dx,*** D D / M M / Y Y<br>☐ **Herpes Simplex** — ***date of dx,*** D D / M M / Y Y<br>☐ **Other,specify:** — ***date of dx,*** D D / M M / Y Y<br>☐ **No documented OIs**
24. History of sexually transmitted infections before or during ART treatment: *(check all that apply)*	☐ **Gonorrhea** ☐ **Chlamydia** ☐ **Syphilis** ☐ **Herpes**<br>☐ **Other (s), specify_______________**<br><br>☐ **No history of sexually transmitted infection**
25. History of other chronic illnesses e.g. Diabetes, Kidney disease, etc before or during ART treatment:	☐ **Yes** ☐ **No** ☐ **Missing**<br>**If yes, please specify: 1. _____________2.__ __________3. ________**
26. Was Patient on Cotrimoxazole (CTX) at start of ART?	☐ **Yes,** ☐ **No** ☐ **Missing**
27. Was Patient on Cotrimoxazole (CTX) at last visit?	☐ **Yes,** ☐ **No** ☐ **Missing**

C. ART REGIMEN

28. If a drug is changed in a regimen (substitution) or if the whole regimen is changed (switch), give date of the change, old and new regimens, and reason(s) for change. Use the below list to pick the reason (s) for change (*complete this section only if there is change in ART regimen, if no change, select no change #7 below*):

1 = Toxicity **2 = pregnancy** **3 = anemia** **4 = active TB**
5 = new medicine available **6 = break in supply of a drug** **7 = other, please specify**

If documentation shows no regimen changes, tick here: ☐ NO REGIMEN CHANGES

Date of change	Old Regimen	New Regimen	Reason(s) for change
D D / M M / Y Y			
D D / M M / Y Y			
D D / M M / Y Y			
D D / M M / Y Y			
D D / M M / Y Y			
D D / M M / Y Y			
D D / M M / Y Y			
D D / M M / Y Y			
D D / M M / Y Y			
D D / M M / Y Y			
D D / M M / Y Y			
D D / M M / Y Y			
D D / M M / Y Y			
D D / M M / Y Y			
D D / M M / Y Y			
D D / M M / Y Y			
D D / M M / Y Y			
D D / M M / Y Y			
D D / M M / Y Y			
D D / M M / Y Y			
D D / M M / Y Y			
D D / M M / Y Y			
D D / M M / Y Y			
D D / M M / Y Y			
D D / M M / Y Y			
D D / M M / Y Y			
D D / M M / Y Y			
D D / M M / Y Y			
D D / M M / Y Y			
D D / M M / Y Y			
D D / M M / Y Y			

(enter appropriate ART 3 letters/letter + number from below)

1a.d4t-3TC-NVP
1b. d4t-3TC-EFV
1c. TDF-3TC-NVP
1d. TDF-3TC-EFV
1e. TDF-FTC-NVP
1f. TDF-FTC-EFV
1g. AZT-3TC-NVP
1h. AZT-3TC-EFV
1j AZT-FTC-NVP
1k. AZT-FTC-EFV

2a.ABC-ddI-SQV/r
2b. TDF-ddI-IDV/r
2c. TDF-3TC-LPV/r
2d. TDF-3TC-IDV/r
2e. TDF-3TC-SQV/r
2f. TDF-FTC-LPV/r
2g. TDF-FTC-IDV/r
2h. TDF-FTC-SQV/r
2i. AZT-TDF-3TC-LPV/r
2j. AZT-TDF-3TC
2k. AZT-FTC-TDF-LPV/r

3a. TDF-ddI-IDV/r
3b. TDF-ddI-LPV/r
3c. ABC-ddI-SQV/r
3d. ABC-ddI-LPV/r

44. Other, specify: / /

**D. ART INTERRUPTIONS AND ADVERSE EVENTS**	
29a. Any history of Stopping ART use?	☐ **If yes, enter 1st stop date:** D D / M M / Y Y<br>☐ **If yes, enter 1st restart date:** D D / M M / Y Y<br>☐ **If stopped a 2nd time, enter 2nd stop date:** D D / M M / Y Y<br>☐ **If stopped a 2nd time, enter 2nd start date:** D D / M M / Y Y<br>☐ **If stopped a 3rd time, enter 3rd stop date:** D D / M M / Y Y<br>☐ **If stopped a 3rd time, enter 3rd start date:** D D / M M / Y Y<br>☐ **No – No history of stopping ART use** , (*skip to question 30*)
29b. If patient **stopped ART**, what was the reason? (*Check all that apply)*	☐ **Developed Active TB** D D / M M / Y Y<br>☐ **Drug toxicity/intolerance** D D / M M / Y Y<br>☐ **IRIS** D D / M M / Y Y<br>☐ **Pregnancy** D D / M M / Y Y<br>☐ **Other, _______________** D D / M M / Y Y<br>☐ **Unknown**
30. Any documented adverse events to ART that patient developed?	☐ **Yes** ☐ **No**<br>***If Yes, check all that apply***: ☐ **Severe rash** ☐ **IRIS**<br>☐ **Anemia** ☐ **Hepatitis** ☐ **Neuropathy**<br>☐ **Other, specify:______________________**

E. FOLLOW UP STATUS

31. List the dates of **<u>ALL</u>** scheduled and actual clinic visits starting from the date patient was started on ART and enter weights, follow-up status for each prior visit (*use page 7 if more space is needed*)
For follow up status use the following codes: **1 = On treatment** **2 = Dead** **3 = Stopped ART**
4 = Lost to follow up **5 = Transferred out** **6 = Restarted ART** **7 = Other, specify____**
8 = Missing

Scheduled date	Actual Visit Date	Weight	Follow up status
D D / M M / Y Y	D D / M M / Y Y	___Kg ☐ Missing	
D D / M M / Y Y	D D / M M / Y Y	___Kg ☐ Missing	
D D / M M / Y Y	D D / M M / Y Y	___Kg ☐ Missing	
D D / M M / Y Y	D D / M M / Y Y	___Kg ☐ Missing	
D D / M M / Y Y	D D / M M / Y Y	___Kg ☐ Missing	
D D / M M / Y Y	D D / M M / Y Y	___Kg ☐ Missing	
D D / M M / Y Y	D D / M M / Y Y	___Kg ☐ Missing	
D D / M M / Y Y	D D / M M / Y Y	___Kg ☐ Missing	
D D / M M / Y Y	D D / M M / Y Y	___Kg ☐ Missing	
D D / M M / Y Y	D D / M M / Y Y	___Kg ☐ Missing	
D D / M M / Y Y	D D / M M / Y Y	___Kg ☐ Missing	
D D / M M / Y Y	D D / M M / Y Y	___Kg ☐ Missing	
D D / M M / Y Y	D D / M M / Y Y	___Kg ☐ Missing	
D D / M M / Y Y	D D / M M / Y Y	___Kg ☐ Missing	

F. COUNSELLING & SUPPORT SERVICES

**Did Patient Receive any of the following?**	
32. Pre-ART counseling	☐ **Yes, *No of times*____** ☐ **No** ☐ **Not collected** ☐ **Missing**
33. Counseling at ART initiation	☐ **Yes, *No of times*____** ☐ **No** ☐ **Not collected** ☐ **Missing**
34. Any adherent counseling during follow-up	☐ **Yes, *No of times*____** ☐ **No** ☐ **Not collected** ☐ **Missing**
35. Does patient attend a support Group?	☐ **Yes** ☐ **No** ☐ **Missing**<br>**If yes, please specify:___________________________**
36. Support services used by the patient since initiation of ART (*check all that apply*)	☐ **Home based care**<br>☐ **Nutritional support**<br>☐ **Community based support groups**<br>☐ **Other, please specify:________________**<br>☐ **Not using any support services**<br>☐ **Missing**
37. Does patient use condoms?	☐ **Always** ☐ **most of the time** ☐ **Occasionally** ☐ **Do not use condoms** ☐ **Not sexually active** ☐ **Not assessed** ☐ **Missing**

G. PHARMACY REGISTER

38. List dates of **<u>ALL</u>** ARV collected, please record date refill given, ARV regimen (use ARV codes from question 28), and number of days for which ARV prescription is given (*use page 8 if more space is needed*)

Date	Regimen	# days of prescription	Date	Regimen	# days of prescription
D D / M M / Y Y			D D / M M / Y Y		
D D / M M / Y Y			D D / M M / Y Y		
D D / M M / Y Y			D D / M M / Y Y		
D D / M M / Y Y			D D / M M / Y Y		
D D / M M / Y Y			D D / M M / Y Y		
D D / M M / Y Y			D D / M M / Y Y		
D D / M M / Y Y			D D / M M / Y Y		
D D / M M / Y Y			D D / M M / Y Y		
D D / M M / Y Y			D D / M M / Y Y		
D D / M M / Y Y			D D / M M / Y Y		

H. LABORTORY RESULTS

39. Please record the test dates and values of **<u>ALL</u>** CD4 **cells/mm³** counts, viral loads (VL) **Copies/dl**, Hemoglobin (Hgb) **g/dl** levels, Alanine aminotransferase (ALT) **U/I**, and Creatinine (Cr) umol/L, for this patient (*use page 9 if more space is needed*)

Visit #	CD4	Date	VL	Date	Hgb	Date	ALT U/I	Date	Cr	Date
1		DD/MM/YY		DD/MM/YY		DD/MM/YY		DD/MM/YY		DD/MM/YY
2		DD/MM/YY		DD/MM/YY		DD/MM/YY		DD/MM/YY		DD/MM/YY
3		DD/MM/YY		DD/MM/YY		DD/MM/YY		DD/MM/YY		DD/MM/YY
4		DD/MM/YY		DD/MM/YY		DD/MM/YY		DD/MM/YY		DD/MM/YY

5		DD/MM/YY		DD/MM/YY		DD/MM/YY		DD/MM/YY		DD/MM/YY
6		DD/MM/YY		DD/MM/YY		DD/MM/YY		DD/MM/YY		DD/MM/YY
7		DD/MM/YY		DD/MM/YY		DD/MM/YY		DD/MM/YY		DD/MM/YY
8		DD/MM/YY		DD/MM/YY		DD/MM/YY		DD/MM/YY		DD/MM/YY
9		DD/MM/YY		DD/MM/YY		DD/MM/YY		DD/MM/YY		DD/MM/YY
10		DD/MM/YY		DD/MM/YY		DD/MM/YY		DD/MM/YY		DD/MM/YY
11		DD/MM/YY		DD/MM/YY		DD/MM/YY		DD/MM/YY		DD/MM/YY
12		DD/MM/YY		DD/MM/YY		DD/MM/YY		DD/MM/YY		DD/MM/YY

**40**. Hepatitis B status for this patient is:	☐ **Antibodies Positive** ☐ **Antigen positive**<br>☐ **Negative** ☐ **Unknown** ☐ **Missing**
**41**. Hepatitis C status for this patient:	☐ **Positive** ☐ **Negative** ☐ **Unknown** ☐ **Missing**<br>**Date of test:** DD/MM/YY

I. KEY OUTCOMES

**42. Date of last clinic visit:**	DD/MM/YY
43. Patient's outcome at the last visit?	☐ **Died,** ***date of death:*** DD/MM/YY<br>☐ **Alive, on ART**<br>☐ **Transferred out,** ***date of transfer out:*** DD/MM/YY<br>☐ **Stopped ART,** ***date of voluntarily stopping care:*** DD/MM/YY<br>☐ **Lost to follow up**
44. If patient died, what was the documented cause of death?	☐ **Pneumonia (not P TB / PCP)** ☐ **Cryptococcal meningitis**<br>☐ **Acute diarrhoea** ☐ **Chronic diarrhoea**<br>☐ **PTB** ☐ **EPTB**<br>☐ **Other, specify:______________** ☐ **Unknown** ☐ **Missing**

Nigeria ART Program Evaluation Data Extraction Tool

If additional space is needed for **question 32,** please use the space below

Continuation from page 4, question 32

**Scheduled date**	**Actual Visit Date**	**Weight**	**Follow up status**
D D / M M / Y Y	D D / M M / Y Y	___Kg ☐ Missing	
D D / M M / Y Y	D D / M M / Y Y	___Kg ☐ Missing	
D D / M M / Y Y	D D / M M / Y Y	___Kg ☐ Missing	
D D / M M / Y Y	D D / M M / Y Y	___Kg ☐ Missing	
D D / M M / Y Y	D D / M M / Y Y	___Kg ☐ Missing	
D D / M M / Y Y	D D / M M / Y Y	___Kg ☐ Missing	
D D / M M / Y Y	D D / M M / Y Y	___Kg ☐ Missing	
D D / M M / Y Y	D D / M M / Y Y	___Kg ☐ Missing	
D D / M M / Y Y	D D / M M / Y Y	___Kg ☐ Missing	
D D / M M / Y Y	D D / M M / Y Y	___Kg ☐ Missing	
D D / M M / Y Y	D D / M M / Y Y	___Kg ☐ Missing	
D D / M M / Y Y	D D / M M / Y Y	___Kg ☐ Missing	
D D / M M / Y Y	D D / M M / Y Y	___Kg ☐ Missing	
D D / M M / Y Y	D D / M M / Y Y	___Kg ☐ Missing	
D D / M M / Y Y	D D / M M / Y Y	___Kg ☐ Missing	
D D / M M / Y Y	D D / M M / Y Y	___Kg ☐ Missing	
D D / M M / Y Y	D D / M M / Y Y	___Kg ☐ Missing	
D D / M M / Y Y	D D / M M / Y Y	___Kg ☐ Missing	
D D / M M / Y Y	D D / M M / Y Y	___Kg ☐ Missing	
D D / M M / Y Y	D D / M M / Y Y	___Kg ☐ Missing	
D D / M M / Y Y	D D / M M / Y Y	___Kg ☐ Missing	
D D / M M / Y Y	D D / M M / Y Y	___Kg ☐ Missing	
D D / M M / Y Y	D D / M M / Y Y	___Kg ☐ Missing	
D D / M M / Y Y	D D / M M / Y Y	___Kg ☐ Missing	
D D / M M / Y Y	D D / M M / Y Y	___Kg ☐ Missing	
D D / M M / Y Y	D D / M M / Y Y	___Kg ☐ Missing	
D D / M M / Y Y	D D / M M / Y Y	___Kg ☐ Missing	
D D / M M / Y Y	D D / M M / Y Y	___Kg ☐ Missing	
D D / M M / Y Y	D D / M M / Y Y	___Kg ☐ Missing	
D D / M M / Y Y	D D / M M / Y Y	___Kg ☐ Missing	
D D / M M / Y Y	D D / M M / Y Y	___Kg ☐ Missing	
D D / M M / Y Y	D D / M M / Y Y	___Kg ☐ Missing	
D D / M M / Y Y	D D / M M / Y Y	___Kg ☐ Missing	
D D / M M / Y Y	D D / M M / Y Y	___Kg ☐ Missing	
D D / M M / Y Y	D D / M M / Y Y	___Kg ☐ Missing	
D D / M M / Y Y	D D / M M / Y Y	___Kg ☐ Missing	
D D / M M / Y Y	D D / M M / Y Y	___Kg ☐ Missing	
D D / M M / Y Y	D D / M M / Y Y	___Kg ☐ Missing	
D D / M M / Y Y	D D / M M / Y Y	___Kg ☐ Missing	
D D / M M / Y Y	D D / M M / Y Y	___Kg ☐ Missing	

Nigeria ART Program Evaluation Data Extraction Tool

If additional space is needed for **question 39,** please use the space below

Continuation from page 5, question 39

Date	Regimen	# days of prescription	Date	Regimen	# days of prescription
D D / M M / Y Y			D D / M M / Y Y		
D D / M M / Y Y			D D / M M / Y Y		
D D / M M / Y Y			D D / M M / Y Y		
D D / M M / Y Y			D D / M M / Y Y		
D D / M M / Y Y			D D / M M / Y Y		
D D / M M / Y Y			D D / M M / Y Y		
D D / M M / Y Y			D D / M M / Y Y		
D D / M M / Y Y			D D / M M / Y Y		
D D / M M / Y Y			D D / M M / Y Y		
D D / M M / Y Y			D D / M M / Y Y		
D D / M M / Y Y			D D / M M / Y Y		
D D / M M / Y Y			D D / M M / Y Y		
D D / M M / Y Y			D D / M M / Y Y		
D D / M M / Y Y			D D / M M / Y Y		
D D / M M / Y Y			D D / M M / Y Y		
D D / M M / Y Y			D D / M M / Y Y		
D D / M M / Y Y			D D / M M / Y Y		
D D / M M / Y Y			D D / M M / Y Y		
D D / M M / Y Y			D D / M M / Y Y		
D D / M M / Y Y			D D / M M / Y Y		
D D / M M / Y Y			D D / M M / Y Y		
D D / M M / Y Y			D D / M M / Y Y		
D D / M M / Y Y			D D / M M / Y Y		
D D / M M / Y Y			D D / M M / Y Y		
D D / M M / Y Y			D D / M M / Y Y		
D D / M M / Y Y			D D / M M / Y Y		
D D / M M / Y Y			D D / M M / Y Y		
D D / M M / Y Y			D D / M M / Y Y		
D D / M M / Y Y			D D / M M / Y Y		
D D / M M / Y Y			D D / M M / Y Y		
D D / M M / Y Y			D D / M M / Y Y		
D D / M M / Y Y			D D / M M / Y Y		
D D / M M / Y Y			D D / M M / Y Y		
D D / M M / Y Y			D D / M M / Y Y		
D D / M M / Y Y			D D / M M / Y Y		
D D / M M / Y Y			D D / M M / Y Y		
D D / M M / Y Y			D D / M M / Y Y		
D D / M M / Y Y			D D / M M / Y Y		
D D / M M / Y Y			D D / M M / Y Y		
D D / M M / Y Y			D D / M M / Y Y		

Nigeria ART Program Evaluation Data Extraction Tool

If additional space is needed for **question 40,** please use the space below

Visit #	CD4	Date	VL	Date	Hgb	Date	ALT U/I	Date	Cr	Date
**13**		DD/MM/YY		DD/MM/YY		DD/MM/YY		DD/MM/YY		DD/MM/YY
**14**		DD/MM/YY		DD/MM/YY		DD/MM/YY		DD/MM/YY		DD/MM/YY
**15**		DD/MM/YY		DD/MM/YY		DD/MM/YY		DD/MM/YY		DD/MM/YY
**16**		DD/MM/YY		DD/MM/YY		DD/MM/YY		DD/MM/YY		DD/MM/YY
**17**		DD/MM/YY		DD/MM/YY		DD/MM/YY		DD/MM/YY		DD/MM/YY
**18**		DD/MM/YY		DD/MM/YY		DD/MM/YY		DD/MM/YY		DD/MM/YY
**19**		DD/MM/YY		DD/MM/YY		DD/MM/YY		DD/MM/YY		DD/MM/YY
**20**		DD/MM/YY		DD/MM/YY		DD/MM/YY		DD/MM/YY		DD/MM/YY
21		DD/MM/YY		DD/MM/YY		DD/MM/YY		DD/MM/YY		DD/MM/YY
22		DD/MM/YY		DD/MM/YY		DD/MM/YY		DD/MM/YY		DD/MM/YY
23		DD/MM/YY		DD/MM/YY		DD/MM/YY		DD/MM/YY		DD/MM/YY
24		DD/MM/YY		DD/MM/YY		DD/MM/YY		DD/MM/YY		DD/MM/YY
25		DD/MM/YY		DD/MM/YY		DD/MM/YY		DD/MM/YY		DD/MM/YY
26		DD/MM/YY		DD/MM/YY		DD/MM/YY		DD/MM/YY		DD/MM/YY
27		DD/MM/YY		DD/MM/YY		DD/MM/YY		DD/MM/YY		DD/MM/YY
28		DD/MM/YY		DD/MM/YY		DD/MM/YY		DD/MM/YY		DD/MM/YY
29		DD/MM/YY		DD/MM/YY		DD/MM/YY		DD/MM/YY		DD/MM/YY
30		DD/MM/YY		DD/MM/YY		DD/MM/YY		DD/MM/YY		DD/MM/YY
31		DD/MM/YY		DD/MM/YY		DD/MM/YY		DD/MM/YY		DD/MM/YY
32		DD/MM/YY		DD/MM/YY		DD/MM/YY		DD/MM/YY		DD/MM/YY
33		DD/MM/YY		DD/MM/YY		DD/MM/YY		DD/MM/YY		DD/MM/YY
34		DD/MM/YY		DD/MM/YY		DD/MM/YY		DD/MM/YY		DD/MM/YY
35		DD/MM/YY		DD/MM/YY		DD/MM/YY		DD/MM/YY		DD/MM/YY
36		DD/MM/YY		DD/MM/YY		DD/MM/YY		DD/MM/YY		DD/MM/YY
37		DD/MM/YY		DD/MM/YY		DD/MM/YY		DD/MM/YY		DD/MM/YY
38		DD/MM/YY		DD/MM/YY		DD/MM/YY		DD/MM/YY		DD/MM/YY
39		DD/MM/YY		DD/MM/YY		DD/MM/YY		DD/MM/YY		DD/MM/YY
40		DD/MM/YY		DD/MM/YY		DD/MM/YY		DD/MM/YY		DD/MM/YY
41		DD/MM/YY		DD/MM/YY		DD/MM/YY		DD/MM/YY		DD/MM/YY
42		DD/MM/YY		DD/MM/YY		DD/MM/YY		DD/MM/YY		DD/MM/YY
43		DD/MM/YY		DD/MM/YY		DD/MM/YY		DD/MM/YY		DD/MM/YY
44		DD/MM/YY		DD/MM/YY		DD/MM/YY		DD/MM/YY		DD/MM/YY
45		DD/MM/YY		DD/MM/YY		DD/MM/YY		DD/MM/YY		DD/MM/YY
46		DD/MM/YY		DD/MM/YY		DD/MM/YY		DD/MM/YY		DD/MM/YY
47		DD/MM/YY		DD/MM/YY		DD/MM/YY		DD/MM/YY		DD/MM/YY
48		DD/MM/YY		DD/MM/YY		DD/MM/YY		DD/MM/YY		DD/MM/YY
49		DD/MM/YY		DD/MM/YY		DD/MM/YY		DD/MM/YY		DD/MM/YY
50		DD/MM/YY		DD/MM/YY		DD/MM/YY		DD/MM/YY		DD/MM/YY
51		DD/MM/YY		DD/MM/YY		DD/MM/YY		DD/MM/YY		DD/MM/YY
52		DD/MM/YY		DD/MM/YY		DD/MM/YY		DD/MM/YY		DD/MM/YY
53		DD/MM/YY		DD/MM/YY		DD/MM/YY		DD/MM/YY		DD/MM/YY
54		DD/MM/YY		DD/MM/YY		DD/MM/YY		DD/MM/YY		DD/MM/YY
55		DD/MM/YY		DD/MM/YY		DD/MM/YY				DD/MM/YY
